# Distal radius fractures in children aged 5–12 years: a Swedish nationwide register-based study of 25 777 patients

**DOI:** 10.1186/s12891-023-06680-8

**Published:** 2023-07-10

**Authors:** Anna Bergkvist, Eva Lundqvist, Evelina Pantzar-Castilla

**Affiliations:** Department of Orthopedics, Orebro University School of Medical Sciences and Orebro University Hospital, Orebro, Sweden

**Keywords:** Epidemiology, Kirschner wire fixation, Pediatric distal radius fracture, Treatment

## Abstract

**Background:**

Distal radius fracture (DRF) is the most common type of fracture in children. There is no clear consensus on primary treatment for complete DRFs. Kirschner wire (K-wire) fixation has been recommended, to avoid the risk of redislocation. However, recent studies have indicated that casting can be sufficient, at least for children with two or more years left to grow. There is no recent study regarding pediatric DRFs and the extent of K-wire fixations in the Swedish population. The purpose of this study was to investigate epidemiology and treatment of pediatric DRFs registered in the Swedish Fracture Register (SFR).

**Methods:**

In this retrospective study, based on data from SFR for children aged 5–12 years with DRF between January 2015 and October 2022, we investigated epidemiology and choice of treatment. Sex, age, type of DRF, treatment, cause and mechanism of injury, were analyzed.

**Results:**

In total, 25,777 patients were included, 7,173 (27%) with complete fractures. Number and peak age of girls vs. boys with fractures were 11,742 (46%), 10 years, and 14,035 (54%), 12 years, respectively. Odds ratio (OR) for a K-wire fixation in girls vs. boys was 0.81 (95% confidence interval (CI) 0.74–0.89, *p* < .001). With age 5 -7 years as reference, OR for age group 8–10 years was 0.88 (95% CI 0.80–0.98 *p* = .019) and OR for age group 11–12 years was 0.81 (95% CI 0.73–0.91 *p* =  < .001.

**Conclusion:**

Casting only was the preferred treatment for all fractures (76%). Boys acquired DRFs more often than girls, with a peak age of 12 years. Younger children and boys with a complete fracture were more likely than older children and girls to receive a K-wire. Further research regarding indications for K-wiring of DRFs in the pediatric population is needed.

## Introduction

Distal radius fracture (DRF) is the most common type of fracture in children (20–30% of all pediatric orthopedic fractures), and most often affects boys [1–5]. The dominant cause of injury is a fall at home or during sports [2–5]. Open physes in children give the potential for re-modulation of fractures, which thus can be managed differently than in adults [3]. A larger fracture displacement can be accepted in younger children and should be considered when choosing a treatment method [6].

DRFs are usually simple and incomplete, treated with a cast for a few weeks, mainly for the pain-relieving effect [3, 7]. A completely displaced fracture warrants evaluation of angulation, alignment, and rotation before an appropriate treatment is chosen [3, 8]. However, most such fractures are treated with closed reduction and casting, or an additional procedure with percutaneous Kirschner wire (K-wire) fixation [6, 7, 9]. Treatment with cast only (without reduction) is also described, but not as common [5]. Usually, fractures treated with closed reduction and casting heal without complication and the child regains normal function [9–11]. There is a risk of redislocation after closed reduction of a complete fracture [4, 9, 12]. Figures as high as 34% have been reported [9]. K-wires were introduced as an addition to closed reduction, to reduce the risk of redislocation [6, 12].

K-wires are inserted after reduction under general anesthesia [13]. Usually, one wire is sufficient for stabilizing the fracture, but in some cases, two or three are needed [4, 11]. There are inherent risks with general anesthesia, and further risks arise from adding foreign material into the bone (risk of pin site infection, wire migration, hypertrophic scarring, neuropraxia, restriction of arm movement, and premature closure of the physis) [4, 6, 12, 14].

Redislocation usually occurs within the first 14 days [9, 15]. The risk factors for redislocation are debated and hard to define [16]. One risk factor is a combined radius and ulna fracture [9]. It has been suggested that a completely displaced DRF entails the greatest risk for redislocation and is therefore an indicator for K-wire fixation [9, 12, 13]. However, a recent study concluded that children with one or more risk factor (both bones fractured, complete displacement of the distal radius, or non-anatomical reduction) are all candidates for primary K-wire fixation [6]. Other studies have suggested that children with two or more years left to grow should be treated with reduction followed by casting [13, 17], and K-wire fixation only when there is unacceptable reduction [8, 17].

The aim of this study was to investigate the epidemiological nature of pediatric DRFs, based on data from the SFR, in children aged 5–12 years regarding population characteristics, type of fracture, cause of injury, and treatment. There is no clear consensus concerning the primary treatment for completely displaced fractures and there are no nationwide guidelines on when to insert a K-wire. The incidence of K-wire usage in complete fractures in Sweden is unknown and therefore of interest and importance to explore.

## Material and methods

### Study design and population

This is a retrospective register study, based on data extracted from the SFR, which was requested after ethical approval (Swedish Ethical Review Authority, reference number 2022–04180-01).

The SFR was established in 2011 and is a nationwide quality register where physicians register fractures in their patients [18]. The register has 100% coverage nationwide [18], but it was not until 2020 that all orthopedic departments in Sweden started registering fractures. In 2015, 36 out of 54 departments did so [19]. In a recent comparison of registered underarm fractures in children between the Swedish Patient Register and the SFR, it was shown that about 60% of all fractures are registered in the SFR [20].

### Study population

The study population consisted of children aged 5–12 years with DRF injured between January 2015 and October 2022 and registered in the SFR.

The lower age limit of 5 years was chosen for two reasons. First, it has been used in previous studies [21]. Further, we considered the fact that the frequency for DRFs has been shown to increase from the age of 5 years [1]. The upper age limit of 12 years was chosen as reasonable to still have open physes in both sexes, since the treatment for fractures with closed physes differs.

### Outcome variables

The following data were extracted and analyzed from the SFR: sex, age, date of injury and treatment, type of DRF (avulsion injury, torus fracture, complete fracture, epiphysiolysis/Salter-Harris type I, II, III, IV or V, prosthetic fracture, fractures with classifications as an adult), cause of injury, mechanism of injury (high- or low-energy trauma), and method of treatment (casting only, reduction without anesthesia, reduction under anesthesia, open reduction, percutaneous K-wire fixation, surgery fixation with plates and/or screws, medullary pinning, extraction of internal or external fixation, reoperation, unknown).

DRFs were divided into complete fracture, torus fracture, and epiphysiolysis fracture, with all Salter-Harris types (I–V) included in the last group. Fractures classified as unknown, adult, prosthetic fractures, or avulsion injuries were excluded.

Causes of injury were divided into assault, biking accident, equestrian, fall between levels, fall from the same level, pedestrian, by motorized vehicle (including all types of motorized vehicles), by object (including several types of injuries caused by different objects), another person, other cause (an external factor other than object), spontaneous/stress fracture, unknown, and unknown transportation.

For methods of treatment, the group “other surgery” included all types of surgical procedures other than open reduction and K-wire fixation. Patients registered for only extraction and reoperation were excluded. For one analysis in the treatment of complete fractures, the groups for casting and both reduction options (with or without anesthesia) were included in the same group. In the analysis of K-wiring ratio for the complete fractures, all other treatment options were compared with K-wiring.

### Statistical methods

All data from the SFR were analyzed with SPSS (version 27.0.1). Categorical variables are presented as numbers and percentages, whereas continuous variables are presented as means and ranges. For the statistical analyses, age was assumed to be a categorical variable and was compared with the others with a chi-squared. A Poisson regression model was used to calculate the incidence rate for K-wire fixation of complete fracture in relation to sex and age, reported as odds ratio (OR) with a 95% confidence interval (CI). A *p*-value of < 0.05 was considered statistically significant.

## Results

### Study cohort

**Fig. 1:**
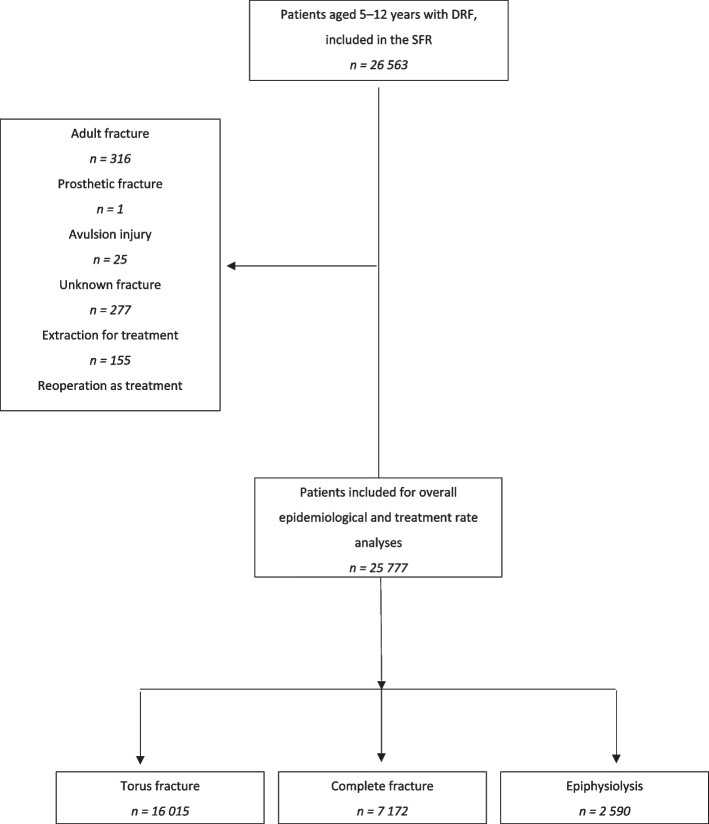
Flowchart of patient selection process

In total, 25 777 patients were included in the epidemiological and statistical analyses, whereas 7 172 patients remained for the analyses of complete fractures (Fig. 1).

### Patient and demographic characteristics

**Fig. 2:**
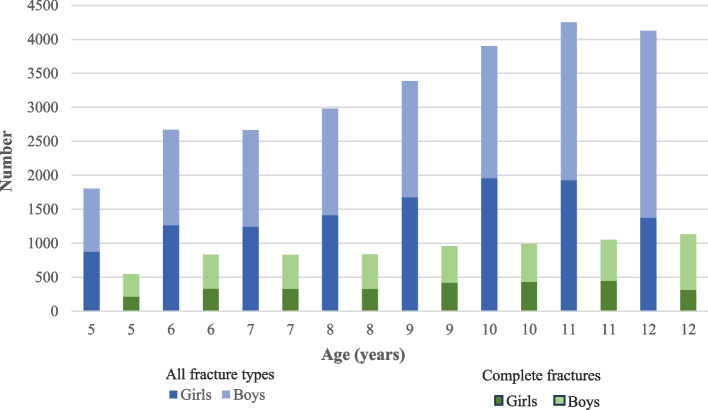
Age distribution between sexes. All included fractures (*N* = 25 777) illustrated in blue and complete fractures (*N* = 7172) in green

Figure 2 shows the age distribution for all included fractures (complete fractures, epiphysiolysis fractures, and torus fractures) and for complete fractures only for girls and boys, with a peak age at 10 years for girls and at 12 years for boys. The overall mean age was 9 years (range 5–12 years). The demographic characteristics are presented in Table 1

**Table 1:** Baseline demographic fracture characteristics

Characteristics	*N* = 25 777 %
**Fracture side**	
Right	42
Left	58
**Mechanism of injury**	
High-energy	5
Low-energy	72
Unknown	23
**Cause of injury**	
Biking accident	9
Equestrian	2
Fall – between levels	30
Fall – same level	42
Motorized vehicle	1
Other cause	1
Other person	2
Other object	8
Unknown	5
**Treatment**	
Casting only	76
K-wire fixation	10
Closed reduction and casting under anesthesia	5
Closed reduction and casting w/o anesthesia	8
Unknown	1

### Fracture types

The fracture classifications (*N* = 25 777) were divided into the three subgroups: complete fractures (28%), epiphysiolysis fractures (10%), and torus fractures (62%) (Table 1).

The overall most commonly reported cause of injury for both sexes was a fall either from the same level (42%) or between levels (30%). Low-energy trauma was the most common mechanism of injury (73%) (Table 1). The prevalence of low-energy vs. high-energy injury was 73% vs. 4% for girls and 71% vs. 5% for boys.

Treatment with casting only was the most common treatment for all included fractures (76%), followed by K-wire fixation (10%). Closed reduction and casting without anesthesia (8%) was more common than under anesthesia (5%) (Table 1). Other treatment than casting only was reported for 36% of the epiphyseiolysis fractures and 5% of the torus fractures (Table 2).

**Table 2:** Cause, mechanism of injury, treatment and gender distribution for children aged 5–12 years divided by fracture type (complete fractures, epifyseolysis and torus fractures)

	Complete fracture **N = 7172** **%**	Epiphysiolysis **N = 2590** **%**	Torus fractur**e** **N = 16 015** **%**
**Cause of injury**			
Biking accident	11	11	8
Equestrian	2	2	1
Fall – between levels	36	29	28
Fall – same level	38	37	45
By motorized vehicle	1	2	1
By object	5	10	10
Another person	2	2	2
Other cause	1	1	1
Unknown	4	5	4
Unknown transportation	1	1	1
**Mechanism of injury**			
High-energy	7	6	3
Low-energy	68	71	75
Unknown	26	23	22
**Treatment**			75
Casting only	43	61	93
K-wire fixation	28	10	1
Closed reduction and casting under anesthesia	10	11	1
Closed reduction and casting w/o anesthesia	15	15	3
Unknown	3	2	2
**Gender**			
Female	24	10	66
Male	32	11	58

For all fracture types in children aged 5–12 years between January 2015 and October 2022, registered in^4^ the SFR.

### Complete fractures

The prevalence of complete fractures compared with other fracture types was 24% for girls and 31% for boys. Complete fractures were more common in older children (12-year-olds, 16%) than younger children (5-year-olds, 8%) (Fig. 2). A fall from the same level (38%) was slightly more common than a fall between levels (36%) (Table 2). Casting with or without reduction was reported for 68% and K-wire fixation for 28% (Table 3).

**Table 3:**
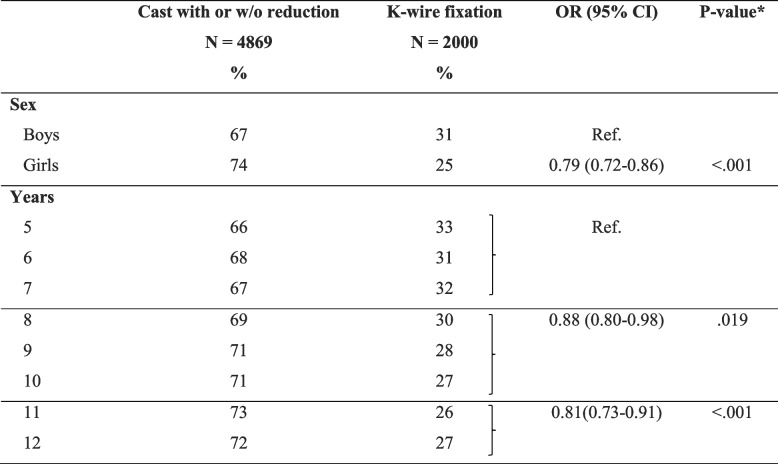
Comparison of treatment options with odds ratio (OR), for boys/girls and age groups with complete fractures

*CI* Confidence interval

*Wald’s chi-squared test for the hypothesis test

In Table 3, the OR for a K-wire fixation is shown and was significantly lower for girls (OR 0.79, 95% CI 0.72–0.86, *p* < 0.001) compared with boys. The OR (with age group 5–7 years as reference) for age group 8–10 years was 0.88 (95% CI 0.80–0.98 *p* = 0.019) and OR for age group 11–12 years was 0.81 (95% CI 0.73–0.91 *p* =  < 0.001).

## Discussion

In this study of children aged 5–12 years with DRF registered in the SFR, the most common fracture was a simple torus fracture and the second most common was a complete fracture. Casting only was the primary treatment most often reported for all fracture types, followed by K-wire fixation. In our results 5% of children registered with a torus fracture received other treatment than casting only, which most likely is a misclassification of the fracture. Younger boys with complete fractures were more likely than girls and older age groups to undergo K-wire fixation.

The peak age was 10 years for girls and 12 years for boys and the mean age was 9 (SD 2) years for both sexes; previous studies show a mean age for children with a DRF ranging between 8 and 13 years [2, 4, 15, 12, 9]. In the Swedish study by Südow et al*.,* based on data from the Swedish National Patient Register for 2005–2013, the mean age was 10 years (range 0–17 years), more specifically 10.7 years for boys and 9.3 years for girls [22].

In this register cohort, boys (54%) more often had a fracture than girls (46%) and this trend grew stronger the older they became, which has been reported previously in both the Swedish-based study of Südow et al*.* [22] and other studies outside of Sweden [1, 2, 9, 12, 15]. Our results also showed that boys were more likely to acquire a complete fracture compared to girls, whereas the prevalence of a torus fracture is higher amongst girls. We did not find other studies that analyzed this specific question.

Multiple studies have shown that the most common cause of injury is a fall [2, 4] and the second most common is an accident during sports, such as biking, skateboarding, or horseback riding [5, 12, 17]. This corresponds to our study results, showing that suffering a fall from either the same or between levels was the most common cause of injury, with the second most common being a biking accident.

We found a statistical significantly higher rate of K-wire fixation in boys than girls. Mahan et al*.* also found a higher surgical treatment rate for boys [23]. Aladraj et al*.* did not report this specifically, but saw a tendency towards boys being more likely to receive a K-wire [14]. Why boys are more likely to undergo a K-wire fixation is unclear. One of the main reasons for a K-wire fixation is to avoid the risk of a redislocation – another is a secondary treatment if the fracture does redislocate [9, 12, 13]. If boys were more prone to redislocation, this could be one explanation. However, previous studies have not shown any significant difference between the sexes [6], indicating that girls are as likely as boys to redislocate and therefore should be as likely as boys to receive a K-wire fixation. Thus, the reason that boys undergo K-wire fixation more often than girls is unclear, and can only be speculated on. Either boys are being overtreated or girls are being undertreated. Are boys presumed to be more physically active and at greater risk of redislocation.

Our results show a decreasing rate of K-wire treatment with each increasing year of age and a significantly lower incidence of K-wire treatment among those 11–12 years old. This differs from the results of Mahan et al*.,* who reported an association between older age and fixation [23]. Their results are therefore more in line with guidelines from previous studies, which suggest that a more conservative approach is more suitable for younger children [23, 24]. The suggestion is that casting after reduction is a suitable first-line treatment for those with at least two years left to grow, unless they have a persistent unacceptable dislocation, in which case K-wire fixation is indicated [15, 24]. We can only speculate as to why the pattern seen in this study differs, but either younger children in Sweden have more severe complete fractures than older children, or younger children undergo a K-wire fixation more often than necessary. Another speculation is that we are more likely to choose primary K-wire fixation if operating resources are limited, to avoid another procedure requiring anesthesia (in the case of redislocation).

Another observation from this study was that the most common treatment, regardless of fracture type, was casting only. This is not surprising, as the most common fracture type was a simple stable torus fracture. Casting only is considered a less favorable option for complete fractures, since it is thought to be insufficient [1, 3, 10, 16, 17] and carries a greater risk of redislocation and other complications, such as malunion [1, 3, 17]. It has been reported that adequate reduction before casting is crucial to prevent redislocation [6, 12, 16]. A K-wire ensures the position of a reduction and it is therefore suggested that a K-wire fixation is more likely to prevent redislocation [12]. However, in the study by Zamzam et al*.,* they did not find a significant correlation between the risk of redislocation and inadequate reduction [9]. On the other hand, more recent studies present satisfying outcomes for remodeling and functional aspects for patients, aged 10 years or younger, with a complete fracture and treated with casting only, which can be one explanation to our results that one third were treated with cast [4, 11, 24, 25]. Still, in a study by Aladraj et al*.* including 176 children, it was concluded that conservative treatment cannot be considered safer than surgical treatment due to the higher complication rate. However, they considered loss of reduction to be a complication and reported superficial pin site infections as common in the operated group [14]. In a survey by the Pediatric Orthopedic Society of North America on the treatment of displaced DRFs, in which 319 surgeons responded, sedated reduction of DRFs was recommended as the primary treatment in completely displaced fractures. However, most surgeons were willing to randomize the treatment of displaced DRFs [5]. This underlines the lack of consensus regarding primary treatment for displaced DRFs.

## Limitations

One inherent limitation is the register-based nature of the study, which depends upon each treating physician registering their patients and treatments, with the risks of incorrectly registered fractures and some patients being missed. Recent reports have shown that around 60% of the total amount of patients are registered, but – more importantly – that there is a skewed distribution of registered fractures between regions [20]. Another factor leading to inconclusive data is that not all regions were active in the register from the beginning – it was only in 2020 that all regions began reporting data there [19]. Some data were misclassified, and some data had been marked as “unknown,” “missing,” or “unspecified”, leading to a smaller sample size in some analyses. However, these can be attributed to human error. Information regarding secondary treatment and complications for any type of treatment has not been received in the data collection process, these factors create a risk of information bias. This is a descriptive study and has not analyzed patient outcomes, which is necessary to evaluate the treatment methods.

## Conclusions

Our study confirms what previously has been shown, that boys acquire fractures more commonly than girls, with a peak age of 12 years and the mean age for both sexes being 9 years within our range of 5–12-year-olds. Casting only is the preferred treatment for all fractures (76%). We found that younger children and boys with complete fractures were more likely to receive a K-wire compared with older age groups and girls, which is not in line with previous studies and thus points out the lack of unity on how to treat these children. Further research regarding indications for K-wiring of DRF in the pediatric population is needed as well as to establish a nationwide consensus.

## Data Availability

The datasets used and/or analyzed during the current study are available from the corresponding author on reasonable request.
